# The involvement of the synaptic vesicle cycle in homocysteine induced neurotoxicity in vitro and in vivo

**DOI:** 10.1038/s41598-025-98306-3

**Published:** 2025-05-29

**Authors:** Meng Wang, Xiaoshan Liang, Keqing Jin, Yinyue Liu, Suhui Luo, Qiang Zhang, Xuan Wang, Zhiping Dong, Xumei zhang

**Affiliations:** 1 Department of Nutrition and Food Science, School of Public Health, Tianjin Medical University, No 22 Qixiangtai Road, Heping District, Tianjin, 300070 China; 2 Tianjin Key Laboratory of Environment, Nutrition and Public Health, Center for International Collaborative Research on Environment, Nutrition and Public Health, Tianjin Medical University, Tianjin, 300070 China; 3 Key Laboratory of Prevention and Control of Major Diseases in the Population, Ministry of Education, Tianjin Medical University, Tianjin, 300070 China; 4 Department of Occupational and Environmental Health, School of Public Health, Tianjin Medical University, Tianjin, 300070 China; 5 Beijing Tiantan Hospital, Capital Medical University, Beijing, 100070 China; 6 NHC Key Laboratory of Hormones and Development, Tianjin Key Laboratory of Metabolic Diseases, Chu Hsien-I Memorial Hospital & Tianjin Institute of Endocrinology, Tianjin Medical University, Tianjin, 300134 China

**Keywords:** Homocysteine, N2a cells, Microarray analysis, Synaptic vesicle cycle, Neurotoxicity, Cell biology, Molecular biology, Neuroscience

## Abstract

Homocysteine (Hcy), a sulfur-containing amino acid derived from methionine, has been shown to be a significant and modifiable risk factor for various neurological disorders, including stroke, Parkinson’s disease, Alzheimer’s disease, and elderly depression. However, there is currently a lack of comprehensive understanding regarding the molecular mechanisms underlying Hcy-induced neurotoxicity. Therefore, this study aimed to establish rat and cell models of Hcy intervention in order to elucidate the underlying mechanism of neurotoxicity. Our research findings demonstrate that Hcy induces depressive - like symptoms in normal Sprague-Dawley rats. Pathological damage and apoptosis were detected in the DG, CA3, and CA1 regions of the hippocampus, along with the cortical area. Moreover, synaptic structural impairment was observed within the hippocampal. Simultaneously, Hcy promotes neuronal apoptosis and LDH leakage in mouse neuroblastoma (N2a) cells. Furthermore, we conducted mRNA microarray analysis to investigate differences in mRNA expressions and utilized Gene ontology (GO) and Kyoto Encyclopedia of Genes and Genomes (KEGG) analysis for gene function annotations in Hcy-treated N2a cells. The results highlighted significant alterations in 457 mRNAs in the Hcy-treated group compared to the Control group. Among the differentially expressed genes (DEGs), a total of 155 were found to be significantly up-regulated, while the remaining 302 were down-regulated. Furthermore, it was observed that four genes (*snap25*,* cplx1*,* slc32a1 and atp6v1e2*) related to the synaptic vesicle cycle exhibited decreased expression in Hcy-treated N2a cells compared to the Control group. The expression levels of these four genes, as well as their corresponding proteins, were subsequently confirmed using RT-qPCR and western blot analysis, respectively. In conclusion, this study shed light on the detrimental impact of hyperhomocysteinemia on the nervous system, particularly with regard to the synaptic vesicle cycle.

**Supplementary Information:**

The online version contains supplementary material available at 10.1038/s41598-025-98306-3.

## Introduction

Homocysteine (Hcy), an excitatory amino acid, has elevated levels when the methionine (Met) cycle metabolism is impaired. This can be due to gene mutations in Hcy - related metabolism or deficiencies in dietary folate, vitamin B6, and B12^1–3^. Epidemiological studies show that hyperhomocysteinemia (HHcy) prevalence is high and region - variable. For example, it’s 73.1% in Iran^4^, 37.2% in China^5^, and 19.1% in Canada^6^. Hyperhomocysteinemia (HHcy), characterized by elevated plasma homocysteine levels, is not only a significant and independent risk factor for cerebrovascular diseases but also strongly associated with various neurodegenerative and mental disorders^7,8^. In the elderly population, higher homocysteine levels are significantly correlated with the severity of cognitive impairment, Alzheimer’s disease (AD), Vascular Dementia (VaD), the severity of neuropsychiatric symptoms, and the degree of functional impairment^9^. Similarly, in children and adolescents, elevated homocysteine levels may lead to depressive symptoms, anxiety disorders, obsessive - compulsive disorder, and difficulties in anger control^10^.

Previous studies have revealed that HHcy may induce neurotoxicity through intricate mechanisms, including the overstimulation of glutamate receptors^11^, participation in oxidative stress reactions^12^, impact on methylation processes^13^, DNA damage^14^, and inhibition of NA^+^/K^+^- ATPase activity^15^. However, further investigation is required to identify the specific mRNAs and signaling pathways that potentially contribute to the neurotoxic effects of Hcy. Employing mRNA expression microarray analysis presents an opportunity to deepen our comprehension of the molecular processes associated with Hcy in the nervous system.

Synapses play a pivotal role in maintaining normal physiological activity and their structural and functional impairment is closely associated with a decline in learning and memory capabilities, cognitive decline, and the onset of depression and anxiety^16^. A growing body of evidence indicates that synaptic dysfunction is a shared characteristic among various neurodegenerative disorders^17^. The controlled regulation of synaptic vesicle exocytosis and subsequent endocytosis, referred to as the synaptic vesicle cycle, is essential for effective neuronal communication and the preservation of neuronal architecture^18^. The study has demonstrated that altered expressions of synaptic proteins, potentially leading to reduced neurotransmission and vesicle replenishment under conditions of prolonged or intense stimulation, play a crucial role in the processes of learning, memory formation, and memory retention^19^. Considering the observed association between elevated levels of Hcy and cognitive impairment, depression, and anxiety, it is hypothesized that the synaptic vesicle cycle may be implicated in the neurotoxic effects of Hcy.

Hence, the principal objective of this study was to explore the potential impacts of homocysteine (Hcy) on nerve cells. Initially, microarray analysis was performed on N2a cells, followed by validation experiments conducted on both N2a cells and rat brain tissues. Subsequently, the study aimed to elucidate the underlying mechanism of action. The findings obtained from this investigation may offer novel insights on the effects of Hcy on the nervous system, as well as identify potential therapeutic targets for the treatment of nervous system disorders exacerbated by elevated Hcy levels.

## Materials and methods

### Animals and experimental design

Adult male Sprague-Dawley (SD) rats (weighing 160–180 g) (grade SPF, Certificate Number SCXK (jing) 2016-0006) was obtained from Beijing Vital River Laboratory Co. Ltd (Beijing, China) and allowed to acclimatize to the laboratory environment for 1 week. The rats were housed under controlled conditions of temperature (22–26 °C) and humidity (40–70%) with a 12/12 h light/dark cycle, and had ad libitum access to food and water. All animal experiments described in this study were conducted in accordance with the Guide for the Care and Use of Laboratory Animals published by the National Institutes of Health (NIH publication no. 80 − 23, revised 1996). The rats were randomly allocated into either the Control group or the Hcy-treated (Hcy) group. Rats in the Hcy group received 2% DL-Hcy solution (5 mL/kg/d; Sigma-Aldrich, St. Louis, MO, USA) for 3 months by intraperitoneal injection^20,21^. All animal protocols (Approval Number: NKYY-DWLL-2021-055) were approved by the Animal Ethical and Welfare Committee (AEWC) of Tianjin Nankai Hospital. All animal protocols (Approval Number: NKYY-DWLL-2021-055) were approved by the Animal Ethical and Welfare Committee (AEWC) of Tianjin Nankai Hospital, and complied with the National Institutes of Health Guidelines for the Care and Use of Laboratory Animals. Our experimental procedures on rats are consistent with the ARRIVE guidelines.

### Open field test (OFT)

OFT was employed to evaluate changes in behavioral activities, specifically locomotor activity and exploratory behavior. To conduct the test, a square cage measuring 50 cm × 50 cm, with walls 40 cm in height, was utilized. The cage contained a black box and a video camera to facilitate data collection. Each rat participant was positioned at the center of the box and given 5 min to freely explore their surroundings. The TM-Vision software (Techman Soft, China) was employed to analyze the total distance traveled and time spent by each rat.

### Sucrose preference test (SPT)

Prior to the commencement of the formal experiment, the rats were provided with two bottles of a 1% sucrose solution for a duration of 24 h. Subsequently, one of the bottles was substituted with pure water, and the rats were allowed to acclimate to this change for an additional 24 h. Following the adaptation procedure, the rats were subjected to a period of food and water deprivation lasting 24 h. Subsequently, the rats were individually housed in cages and granted unrestricted access to two bottles, one containing the aforementioned sucrose solution and the other containing pure water. After a span of 24 h, the quantities of sucrose solution and water consumed by the rats were meticulously recorded. The sucrose preference percentage was then calculated using the following formula: Sucrose preference (%) = sucrose consumption / (sucrose consumption + pure water consumption) × 100%.

### Euthanasia

One day after the conclusion of behavior testing, animals were anesthetized with isofluorane and brains were rapidly dissected and frozen.

### Hematoxylin-eosin (HE) staining

Following OFT, the rats were subjected to anesthesia using an isoflurane vaporizer. Subsequently, the brains were promptly removed, fixed with 4% paraformaldehyde solution, and then embedded in paraffin. Serial sections measuring 6 μm in thickness were then prepared (*n* = 4 per group). These sections were subjected to dewaxing in xylene and rehydration in graded alcohols, followed by staining with hematoxylin and eosin. Subsequently, the sections underwent dehydration using alcohol gradients and xylene, and were blocked using neutral gum. The resulting pathological changes in the brain tissues were then observed using a light microscope (IX81; Olympus, Tokyo, Japan).

### Terminal-deoxynucleotidyl Transferase (TdT)-mediated dUTP nick end labeling (TUNEL) staining

Neuronal apoptosis was detected using the terminal-deoxynucleotidyl Transferase (TdT)-mediated dUTP nick end labeling (TUNEL) staining method in strict accordance with the manufacturer’s instructions (DeadEndTM Fluorometric TUNEL System, G3250, Promega, Madison, WI, USA). Initially, the tissue sections underwent a series of pre-treatment steps. They were first deparaffinized in xylene, then dehydrated with gradient-concentration alcohol solutions. Subsequently, the sections were fixed in a 4% paraformaldehyde solution. After that, the TUNEL reaction was carried out by incubating the sections with the TUNEL reaction mixture in a 37 °C incubator for 60 min. Finally, the stained sections were mounted with an anti - fluorescence quenching blocking buffer and visualized under an inverted fluorescence microscope (OlympusIX81, Olympus, Tokyo, Japan) for analysis of neuronal apoptosis.

### Transmission electron microscopy

For hippocampal synaptic ultrastructure analysis via transmission electron microscopy (TEM), rat hippocampal tissue was cut into 1 mm^3^ fragments and post - fixed in 2.5% glutaraldehyde. Samples were then cut, stained with 2% uranyl acetate, dehydrated in gradient - concentration acetone, and embedded in epoxy resin. After sectioning into 70–90 nm slices, they were double - stained with lead citrate and uranyl acetate for enhanced contrast. Imaging was performed using a TEM (HT7700, Hitachi High - Tech). Synapses were counted manually as per previous reports^22^. The postsynaptic density width was quantitatively analyzed with Image J 1.8.0 software, following established methods, to explore synaptic function and potential neurological changes^23^.

### Cell cultures and treatment

The N2a cell line, obtained from Huaxi Xu at Biomedical Research, Xiamen University, Xiamen, China, was cultured in Dulbecco’s Modification of Eagle’s medium (DMEM; Gibco, USA) supplemented with 10% fetal bovine serum (FBS; Gibco) at 37 °C in a humidified atmosphere containing 5% CO_2_ for a duration of 5 days. The cells were divided into two groups: the Control group and the Hcy-treated (Hcy) group. In the Hcy group, DL-Hcy (500 µM; Sigma, USA) was added to the cell culture medium^24^. The culture medium was refreshed every 2 days.

### Cell viability assays by cell counting kit-8 (CCK-8)

The cell viability was assessed using the CCK-8 assay in accordance with the manufacturer’s instructions. The cells were initially cultured in 96 well plates at a density of 1 × 10^4^ cells per well. Following the intervention, 10 µL of CCK-8 reagent (Bioss Biotech, China) was added to each well and incubated for 0.5 h at 37 °C. The optical density (OD) value at 450 nm was subsequently measured using a microplate reader (ELX800 Ultra Microplate Reader, BioTek Instruments Inc., Winooski, VT, USA).

### Evaluation of lactate dehydrogenase (LDH) release

LDH released into the cell culture supernatants was determined using the LDH cytotoxicity detection kit, following the manufacturer’s instructions (Nanjing Jiancheng Biological Engineering Institute, China). The OD value of each well was measured at 490 nm using a microplate reader (ELX800 Ultra Microplate Reader, BioTek Instruments Inc., Winooski, VT, USA). The percentage of LDH release was calculated using the following equation: LDH release (%) = (Experimental LDH release − spontaneous LDH release) / maximum LDH release.

### Apoptosis analysis

N2a cell apoptosis was assessed using an Annexin V-AbFluorTM 488/PI apoptosis detection kit, following the manufacturer’s instructions. Briefly, N2a cells were collected and rinsed twice with ice-cold PBS. Subsequently, 1 × 10^6^ cells were suspended in 100 µL of 1× Annexin V binding buffer and incubated with 5 µL Annexin V-FITC and 2 µL PI at room temperature in a light-restricted environment for 15 min. Flow cytometry analysis (Becton-Dickinson, San Diego, California, USA) was performed to examine the samples. The apoptotic rate was determined as the percentage of cells in the Q2 + Q3 quadrants.

### RNA purification and Preparation for microarray analysis

The N2a cells from the Control and Hcy groups were washed three times with ice-cold phosphate- buffered saline (PBS) and homogenized using 500 µL Trizol reagent (Gibco BRL, Rockville, MD). Then the samples were collected and pipetted into 1.5-ml microcentrifuge tubes for the following experiments. The mRNA was extracted from the collected RNA samples using a mirVanaTM RNA Isolation Kit (Invitrogen, Karlsruhe, Germany) according to the manufacturer’s instructions. The mRNA from each sample was quantified at 260 nm using a NanoDrop ND-1000 instrument, and the optical density (OD) was determined by the 260/280 ratio. The OD values of the RNA were between 1.80 and 2.20. The mRNA samples were then frozen and stored at -80 ºC.

### mRNA chip microarray analysis

The mRNA expression analysis of the RNA samples was conducted utilizing the Agilent Mouse 8 × 60 K microarray. The microarray hybridization, data collection, and analysis were performed by Oebiotech Biotechnology Corp (Shanghai, China). A fold-change threshold of ≥ 2.0 was employed to determine the upregulated and downregulated mRNAs.

### Functional and pathway enrichment analysis of differently expressed genes (DEGs)

The Gene Ontology (GO) method was utilized to annotate genes and gain insight into potential biological processes, specifically biological processes (BP), cellular component (CC), and molecular function (MF). GO terms with corrected *P* values < 0.05 were deemed significantly enriched in the genes that exhibited differential expression. The Kyoto Encyclopedia of Genes and Genomes (KEGG) pathway was employed to present the annotation and visualization of gene functions (http://www.genome.jp/kegg/)^25–27^.

### Western blot

The brain hippocampus tissues were homogenized in RIPA buffer (Shandong Sparkjade Biotechnology Co., Ltd) supplemented with the protease inhibitor phenylmethylsulfonyl fluoride (PMSF) for a duration of 30 min. Following centrifugation (14,000 rpm for 15 min at 4 °C), the resulting supernatant was collected. The protein concentration was determined using a BCA protein assay kit (Beyotime Biotechnology). Equal amounts of protein (30 µg) from each sample were then separated by SDS-PAGE and subsequently transferred to Polyvinylidene Fluoride (PVDF) membranes (Millipore Corporation, USA). To determine the molecular weights of the proteins, a pre-stained protein molecular weight marker (C05-09002, Bioss, China) was employed. After visualizing the protein bands with the marker as a reference, the relevant bands were excised for further steps. After blocking with 5% nonfat milk, the membranes were incubated with primary antibodies at 4 °C overnight. The primary antibodies used were as follows: anti-SNAP25 (#ab65783, Abcam, USA), anti-Complexin (#28070, Cell Signaling Technology, USA), anti-VGAT (bs-10958R, Bioss, China), anti-ATP6V1E2 (13235-1-AP, SanYing, China). After being washed with the Tris-buffered saline-Tween 20 (TBST) buffer three times, the PVDF membranes were incubated with appropriate horse radish peroxidase (HRP)-conjugated secondary antibodies. Then the proteins were detected with an ECL detection solution (Millipore Corporation) and system (Bio-Rad, USA). The intensity of the protein bands was quantified with ImageJ 1.8.0 software (National Institutes of Health, USA) and then normalized to that of β-actin.

### Reverse transcription quantitative PCR (RT-qPCR)

Total RNA was extracted from N2a cell lines using Trizol reagent (Sangon Biotech (Shanghai) Co., Ltd. China). Subsequently, 1 µg of total RNA was reverse transcribed in a 20 µL reaction system. RT-qPCR was conducted utilizing 10 µL of the reaction mixture, which consisted of 100 ng of cDNA, 2× SYBR green (Sparkjade Biotechnology Co., China), 10 µM of forward and reverse primers, and ddH_2_O. The amplification was carried out using Roche480 (Roche, Switzerland), with an initial denaturation step at 95 °C for 30 s, followed by 40 cycles at 95 °C for 15 s and 60 °C for 15 s. All data were subjected to relative quantification using the 2^−ΔΔCt^ method. Each sample was analyzed in triplicate, and at least three independent runs were performed. The specific primers employed for RT-qPCR are listed in Table 1.

**Table 1:** Primers used for RT-qPCR.

Gene	Primer sequence (5’– 3’)
*snap25* -Forward	ATCTGGCGATTCTGGGTGT
*snap25*-Reverse	CGGGAAAATGAAATGGATGA
*cplx1*- Forward	AGTTCGTGATGAAACAAGCCC
*cplx1*-Reverse	TCTTCCTCCTTCTTAGCAGCA
*atp6v1e2*- Forward	CACCCTGGAAACTCTGTTCTAC
*atp6v1e2*-Reverse	CTGCACGTCTATGTCAGTCAG
*slc32a1*- Forward	ACCTCCGTGTCCAACAAGTC
*slc32a1*- Reverse	TCAAAGTCGAGATCGTCGCAG
*gapdh* - Forward	AACTTTGGCATTGTGGAAGG
*gapdh -* Reverse	ACACATTGGGGGTAGGAACA

### Statistical analysis

All data are presented as the mean ± standard deviation (SD). Statistical significance was tested by Student’s t test, and *p* value < 0.05 was considered to be statistically significant.

## Results

### Hcy induced depressive-like symptoms and neural cell injury and apoptosis in SD rats

**Fig. 1:**
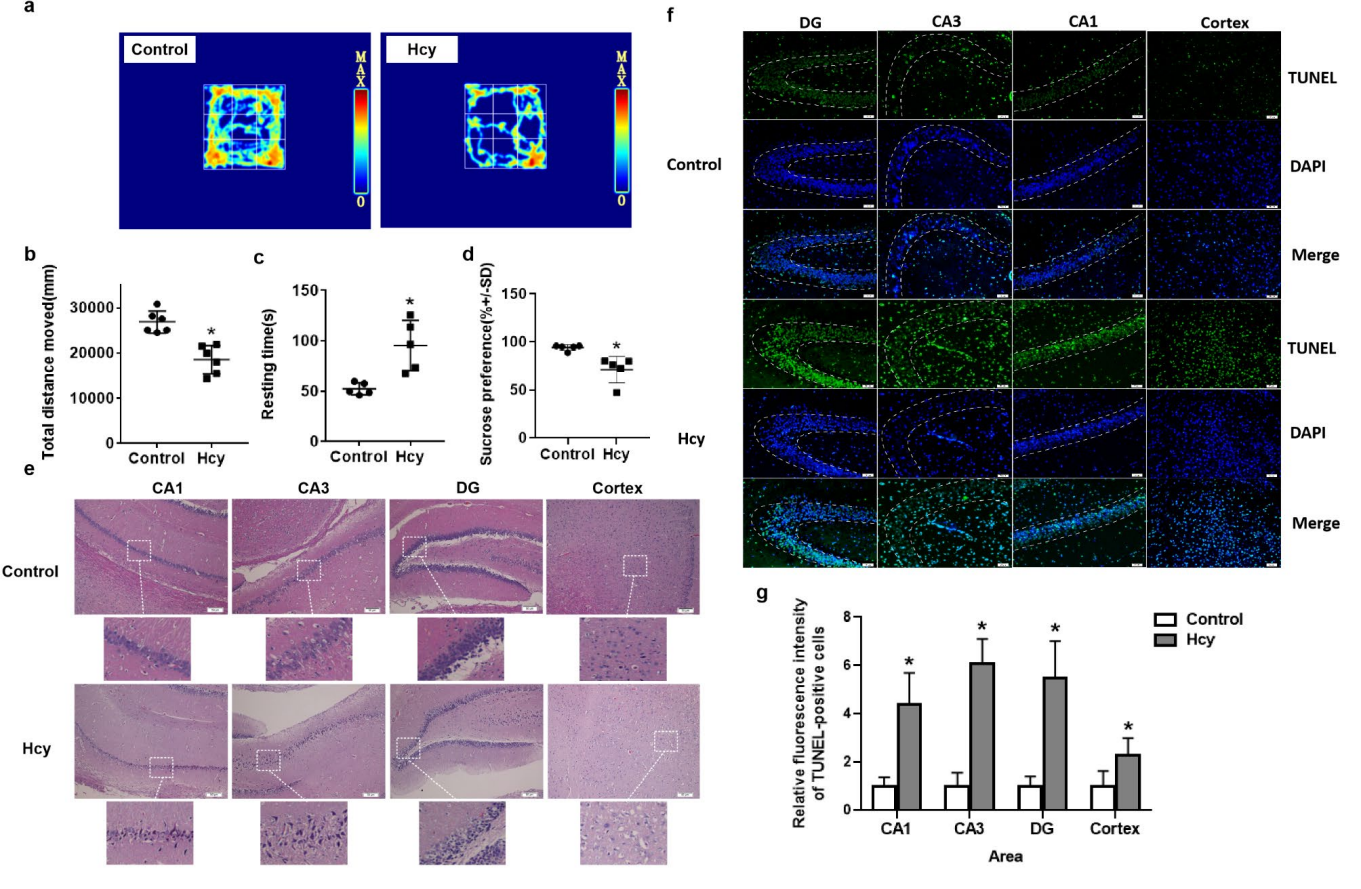
Hcy induced depressive-like symptoms and neural cell injury and apoptosis in SD rats. (**a**) Representative images of OFT. (**b**) The scatter plots depicted the extent of rats’ unrestricted mobility during the OFT. (**c**) Scatter plot depicted the resting time of rats during the OFT. (**d**) Scatter plots depicted the sucrose consumption ratio in SPT. (**e**) The histological outcomes of hematoxylin - eosin staining was examined in the hippocampus (CA1, CA3 and DG) and Cortex regions. (**f**) Representative immunofluorescence images of terminal dexynucleotidyl transferase (TdT)-mediated dUTP nick end labeling (TUNEL)-positive cells (green) and DAPI (blue) in rat brain tissue. White dot lines represent the borders of CA3, DG, and CA1 regions. (**g**) The bar graphs represent the relative fluorescence intensity of TUNEL-positive cells per section. Data were expressed as mean ± SD, *n* = 6 per group, ^*^p < 0.05 compared with Control group.

In order to assess depressive-like behaviors, two behavioral tests were conducted, namely the open field test (OFT) and the sucrose preference test (SPT) (Fig. 1). The OFT was employed to evaluate the spontaneous exploratory behavior of rats in an unfamiliar environment within a 5 min timeframe. The impact of Hcy on the total distance moved and rearing time in the OFT was depicted in Fig. 1a, b, c. Rats in the Hcy-treated group exhibited a decrease in total distance moved (t = 4.741, df = 8, *p* = 0.001) and an increase in resting time compared to the Control group (t = -3.741, df = 8, *p* = 0.006). The results of SPT indicated a noteworthy decrease in preference for sucrose solution among rats subjected to Hcy treatment in comparison to the Control group (Fig. 1d, t = 3.662, df = 8, *p* = 0.006).

Additionally, following the OFT, HE staining and TUNEL staining were respectively employed to assess the morphological features and apoptosis of neural cells in the hippocampal and cortical regions. In the Control group, the neural cells exhibited a well-defined circular shape, intact cytoplasm, and a clearly visible nucleus. Conversely, in the Hcy-treated group, the neurons displayed disorganized arrangement, indistinct features, absence of a distinct cell boundary, and a condensed or significantly reduced nucleus. The extent of cellular damage was more pronounced in the Hcy-treated group when compared to the control group (Fig. 1e). TUNEL staining results showed that that the relative fluorescence intensity of TUNEL positive cells was increased in the Hcy group, compared to the Control group (Fig. 1f, g, *p* < 0.05).

### The exposure to Hcy resulted in cellular damage of N2a cells

**Fig. 2:**
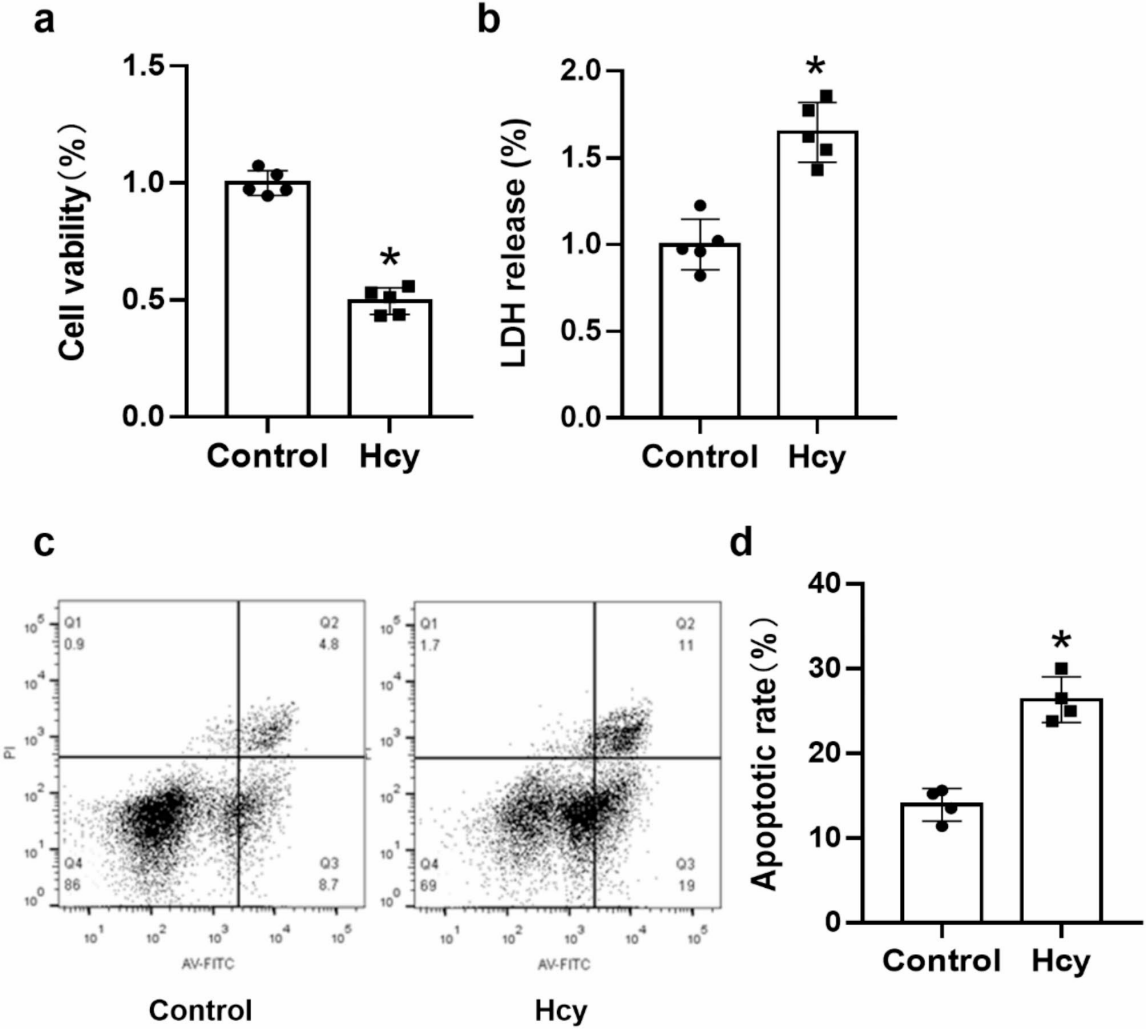
The toxic effect of Hcy on N2a cells in vitro. Cell viability (**a**) and LDH release (**b**) were evaluated by CCK-8 assay and LDH assay, respectively. The data were expressed as the mean ± SD, *n* = 5 per group. ^*^p < 0.05. (**c**) Flow cytometry plots were used to illustrate the apoptosis of N2a cells in both the Control and Hcy groups. (**d**) Scatter plots were used to depict the apoptosis ratio of N2a cells. ^*^p < 0.05 compared with Control group.

Next, we evaluated the toxicity of Hcy to neuronal N2a cells. The impact of Hcy on cell viability and its cytotoxicity were determined through the utilization of CCK-8 and LDH kits, respectively. The corresponding values are presented in the histogram displayed in Fig. 2a, b. In comparison to the Control group, the viability of N2a neuronal cells in the Hcy group exhibited a significant reduction (t = 14.503, df = 8, *p* < 0.001), while LDH leakage in the N2a neuronal cells displayed a significant increase (t = -6.392, df = 8, *p* < 0.001). Additionally, conditioned culture with Hcy resulted in significantly increased apoptosis (t = -7.517, df = 6, *p* < 0.001), as shown in Fig. 2c, d.

### The identification and examination of specific genes targeted by Hcy in N2a cells

**Fig. 3:**
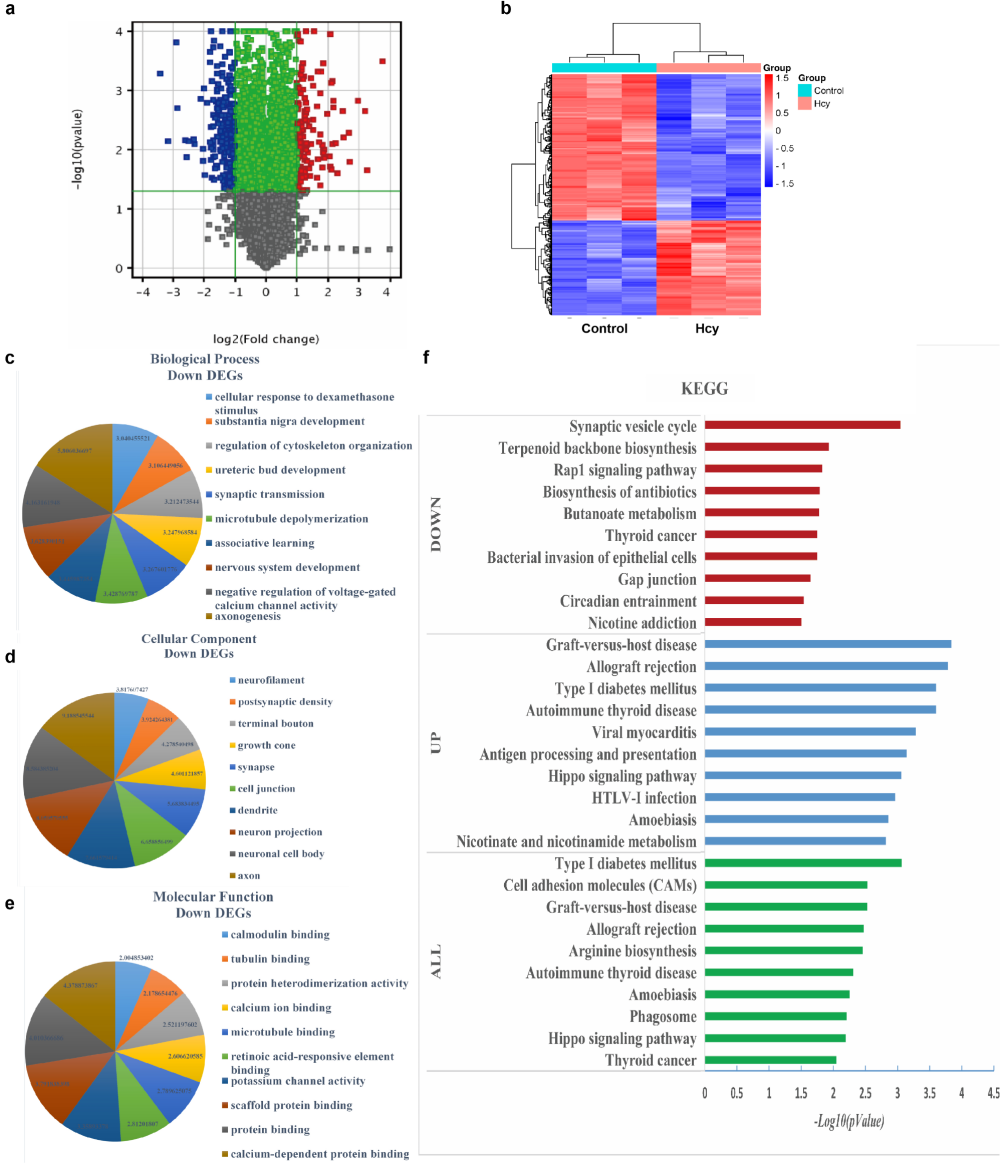
The mRNA chip microarray analysis in N2a cells. (**a**) Volcano plot of microarray data. Horizontal axis represented the log2-fold change, and vertical axis represented the adjusted *p* value. Red plots indicate down-regulated genes, blue plots indicated up-regulated genes, black and green plots indicated genes with no significant difference. (**b**) Hierarchical cluster heat map from the common DEGs. Each column represented a sample, while each row represented a gene. The expression values were depicted using shades of red and blue, indicating expression above and below the median value, respectively, across all samples. Data were represented as log2 fold change, *n* = 3 per group. (**c**) The GO analysis was conducted to categorize DEGs into distinct groups based on the biological process (BP) theme. (**d**) The DEGs were subjected to GO analysis, resulting in their classification into various groups according to the cellular component (CC) theme. (**e**) The GO analysis was performed to classify DEGs into different groups based on the molecular function (MF) theme. (**f**) The differentially expressed genes (DEGs) were subjected to KEGG pathway analysis^25–27^. The -Log10 values of down-regulated DEGs were represented by red pillars, while the -Log10 values of up-regulated DEGs were represented by blue pillars. The -Log10 values of all DEGs were represented by green pillars.

A total of 457 differentially expressed genes (DEGs) were identified between the Control and Hcy-treated groups by applying the cutoff criteria of |log FC| > 2.0. Among these DEGs, 155 were up-regulated and 302 were down-regulated, as visually depicted in the volcano plot (Fig. 3a). To visually represent the expression patterns of these distinguishable genes in the experimental samples, a heat map was generated (Fig. 3b). The hierarchical cluster analysis indicated that the difference spectrum between the Hcy and Control groups is reliable. Additionally, Table 2 presents the top 10 hub genes.

The DEGs were submitted to the DAVID database to evaluate their Gene Ontology (GO) functions (Fig. 3c, d, e). The results of the GO analysis revealed that the down-regulated DEGs in cells treated with Hcy were notably enriched in biological processes related to axonogenesis, voltage-gated calcium channel, and nervous system development. Furthermore, the analysis of the cell component revealed that the DEGs in cells treated with Hcy were primarily implicated in the axon and neuronal cell body. Additionally, the molecular function analysis indicated that the genes in the Hcy group were predominantly associated with calcium-dependent protein binding, protein binding, and scaffold protein binding.

The DAVID online tool was utilized to perform KEGG enrichment analysis on a total of 457 differentially expressed genes (DEGs). The results of the KEGG pathway analysis indicated that all the DEGs, which were regulated in response to Hcy treatment, exhibited significant enrichment in ten KEGG pathways, including type I diabetes mellitus, cell adhesion molecules (CAMs), and graft-versus-host disease (Fig. 3f). Furthermore, the up-regulated DEGs demonstrated significant enrichment in KEGG pathways associated with graft-versus-host disease, allograft rejection, and type I diabetes mellitus. Conversely, the down-regulated DEGs exhibited significant enrichment in KEGG pathways related to the synaptic vesicle cycle, terpenoid backbone biosynthesis and nicotine addiction. It was noteworthy that the synaptic vesicle cycle exhibited a remarkable level of significance among the down-regulated DEGs in relation to Hcy. Moreover, *snap25*, *cplx1*, *slc32a1* and *atp6v1e2* were identified as the hub genes within the synaptic vesicle cycle (Table 2).

**Table 2:** The top 10 down-regulated hub genes in Hcy-treated N2a cells.

Gene Symbol	*p*	|log FC|	Regulation	Strand	Genomic Coordinates
*snap25*	6.68E-04	2.2037911	down	+	chr3:124893776–124,893,835
*slc32a1*	2.59E-03	3.2781937	down	+	chr3:149347048–149,347,107
*gad2*	9.36E-03	3.433208	down	+	chr17:96321562–96,321,621
*tac1*	2.01E-03	7.2841682	down	+	chr4:32657551–32,657,610
*calb2*	1.43E-03	4.2882257	down	−	chr19:40023132 − 40,023,073
*sst*	3.14E-02	2.0621727	down	+	chr11:79126157–79,126,216
*cck*	5.13E-03	2.335657	down	−	chr8:126492700 − 126,492,642
*drd2*	1.97E-03	2.6284552	down	+	chr8:52707670–52,707,729
*synpr*	7.47E-03	3.525207	down	−	chr15:13135390 − 13,135,331
*gap43*	1.76E-03	2.139264	down	+	chr11:60083320–60,083,379

FC, fold-change. E, exponent.

### Hcy reduced the expression of synaptic cycle-related mRNAs and proteins in N2a cells

**Fig. 4:**
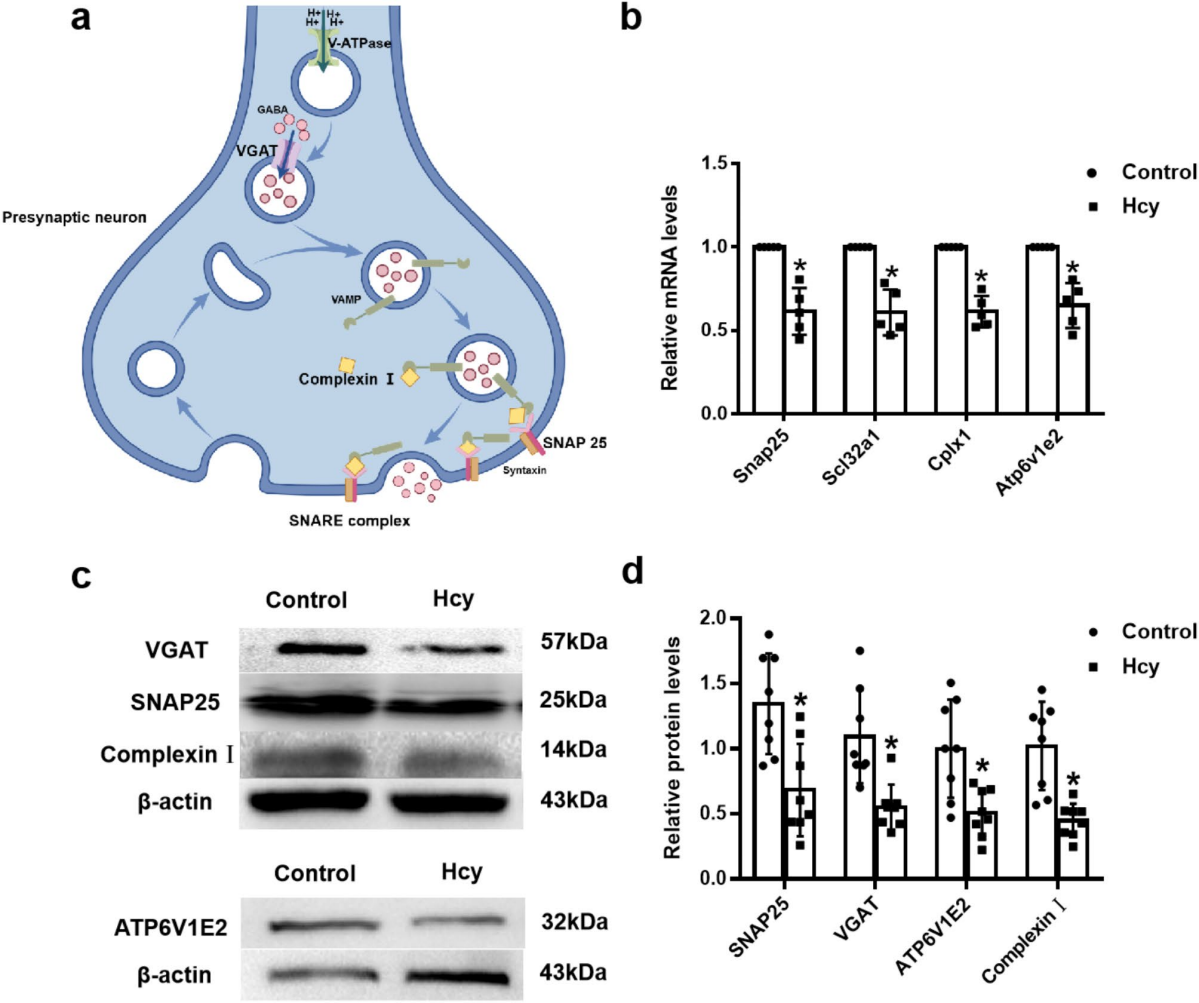
Hcy reduced the expressions of synaptic cycle-related mRNAs and proteins. (**a**) This diagram illustrates mechanisms of synaptic vesicle cycling, including neurotransmitters transport, storage, and release. Initially, the V-ATPase (proton pump, encoded by *atp6v1e2*) uses ATP to pump protons (H^+^) into the vesicle, generating a proton gradient across the vesicle membrane. This proton gradient drives the VGAT (GABA transporter, encoded by *slc32a1*), which transports GABA from the cytosol into the synaptic vesicle for storage. Upon the arrival of a neural signal, Complexin I (encoded by *cplx1*) interacts with the SNARE complex, regulating the approach of the vesicle to the presynaptic membrane. SNAP-25 (encoded by *snap25*), a core component of the SNARE complex, assembles with syntaxin and VAMP to form the SNARE complex, facilitating fusion between the vesicle membrane and the presynaptic membrane. The interaction between Complexin I and SNAP-25 is crucial in ensuring that vesicle fusion occurs at the appropriate time, thereby enabling the precise release of GABA into the synaptic cleft. (**b**) Bar graphs showing relative *snap25*,* cplx1*,* slc32a1* and *atp6v1e2* mRNA levels (normalized to GAPDH mRNA levels) determined by quantitative RT-PCR, *n* = 5 per group, ^*^p < 0.05 compared with Control group. (**c**), (**d**) Representative western blotting of SNAP25, VGAT, ATP6V1E2, Complexin Ι and β-actin. Bar graphs showed the semiquantitative levels of SNAP25, VGAT, ATP6V1E2 and Complexin Ι normalized to β-actin as determined by band density analysis respectively. Data were expressed as mean ± SD, *n* = 8 per group, ^*^p < 0.05 compared with Control group.

A mechanism diagram (Fig. 4a) illustrates the roles of four key genes (*snap25*,* cplx1*,* slc32a1* and *atp6v1e2*) in synaptic vesicle recycling. To validate the results of the transcriptome sequencing, transcripts of four key DEGs associated with the enriched KEGG pathways were detected using RT-qPCR. The results showed that the expression *snap25*,* cplx1*,* slc32a1* and *atp6v1e2* were significantly down-regulated in the Hcy group compared to the Control group (Fig. 4b, t = 6.138, df = 8, *p* < 0.001; t = 9.178, df = 8, *p* < 0.001; t = 6.388, df = 8, *p* < 0.001; t = 5.790, df = 8, *p* < 0.001). The gene expression changes identified by the microarray results were consistent with those analyzed by RT-qPCR. Additionally, these four genes-related protein expression levels were detected by western blotting. As shown in Fig. 4c, d, the protein expression levels of SNAP25, VGAT, ATP6V1E2 and Complexin Ι were decreased after treatment with Hcy (t = 3.566, df = 14, *p* = 0.003; t = 3.854, df = 14, *p* = 0.002; t = 3.336, df = 14, *p* = 0.005; t = 4.463, df = 14, *p* < 0.001).

### Hcy damaged the synaptic ultrastructure and down - regulated the expression of proteins involved in the synaptic vesicle cycle in rat brain tissue

**Fig. 5:**
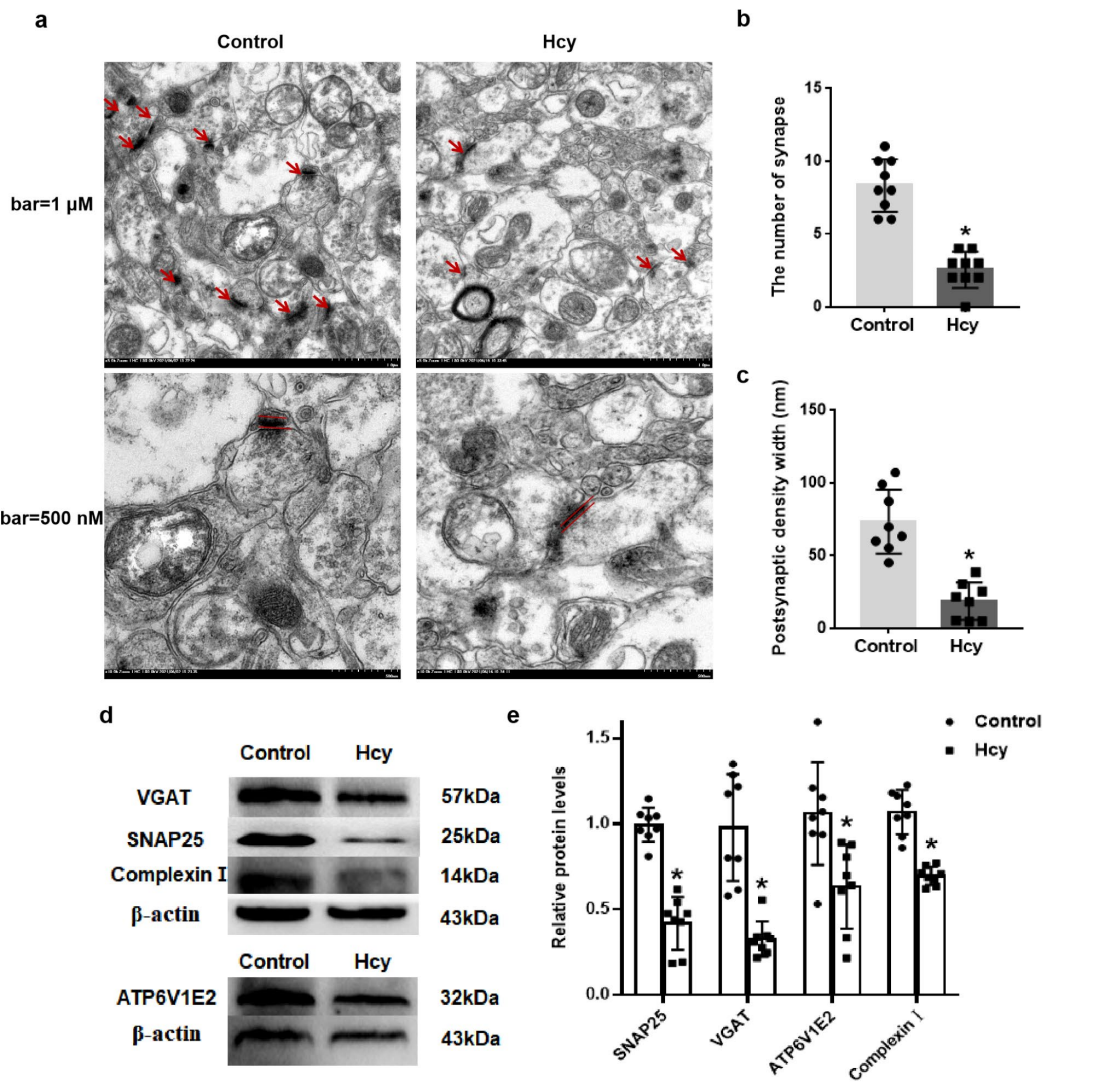
Hcy damaged the synaptic ultrastructure and down - regulated the expression of proteins involved in the synaptic vesicle cycle in rat brain tissue. (**a**) Representative TEM images of synapse in hippocampus. (Scale bar = 1 μm and 500 nm, *n* = 3 per group). Arrows indicated synapses. (**b**) Bar graph showed the number of synapses. Results from quantitative analysis of (**c**) postsynaptic density width. (**d**) Representative western blot bands of VGAT, SNAP25, ATP6V1E2, Complexin Ι and β-actin. (**e**) Semiquantitative analysis of the western blot. The protein levels of SNAP25, VGAT, ATP6V1E2, and Complexin Ι were normalized to β-actin, respectively. Data were expressed as mean ± SD, *n* = 8 per group, ^*^p < 0.05 compared with the Control group.

In the present study, transmission electron microscopy (TEM) was employed to visualize the ultrastructure of synapses within the hippocampus (Fig. 5a). Subsequently, the quantity of synapses and the width of the postsynaptic density were meticulously counted and measured. The abundant number of synapses as well as complete and clear synaptic ultrastructure were detected in the control group. In contrast, the Hcy group exhibited a statistically significant decrease in both the synapse number and the postsynaptic density thickness (Fig. 5b, c, *p* < 0.05).

Western blotting was applied to verify the effect of Hcy on the expressions of four specific proteins associated with the synaptic vesicle cycle in the brain tissue of rats (Fig. 5d, e). The results showed that the SNAP25, Complexin Ι, ATP6V1E2 and VGAT protein levels were significantly decreased in the Hcy group, compared to the Control group (t = 8.818, df = 14, *p* < 0.001; t = 7.593, df = 14, *p* < 0.001; t = 3.108, df = 14, *p* = 0.008; t = 5.578, df = 14, *p* < 0.001).

## Discussion

There exists a substantial body of literature that substantiates the notion that elevated levels of Hcy beyond physiological thresholds pose a risk for cerebrovascular and neurodegenerative diseases. Indeed, epidemiological studies and clinical investigations have established a correlation between hyperhomocysteinemia and conditions such as stroke^28^, epilepsy^29^, Parkinson’s disease^30^, dementia^31,32^, and depression^33^. Simultaneously, the experimental data has provided substantial evidence indicating that Hcy can potentially induce cellular damage through various intricate mechanisms, such as the generation of reactive oxygen species, heightened lipid peroxidation, induced nitrosative stress, and compromised production of the antioxidant glutathione peroxidase. Nevertheless, the underlying mechanism of neurotoxicity induced by exposure to Hcy has not been fully elucidated thus far. This study reveals novel findings indicating that mRNAs associated with the synaptic vesicle cycle are significant molecular targets in the context of Hcy-induced neurotoxicity. The detrimental effects of Hcy on nerve cells are attributed as the disruption of the synaptic vesicle cycle. The present analyses provide a fresh perspective on the molecular mechanisms underlying Hcy-induced neurotoxicity, potentially laying the groundwork a foundation for future investigations.

The relationship between Hcy and depression has been consistently observed in numerous population studies. Aishwarya et al.. revealed a significant association between elevated Hcy levels and postpartum depression in women, both within 24–48 h and 6 weeks after childbirth^34^. Similarly, Esnafoglu et al.. discovered a positive correlation between the severity of depression and Hcy levels in children and adolescents^35^. A longitudinal study conducted by the Cooper center revealed that an elevated level of Hcy can significantly augment the possibility of experiencing symptoms of depression among a cohort of 11,757 participants ranging in age from 20 to 90 years^36^. In the current investigation, we observed that Hcy has the potential to diminish both sucrose consumption and exercise activity in OFT conducted on rats. Furthermore, following stimulation with Hcy, N2a cells exhibited an escalated release of LDH and an increased rate of apoptosis. These findings strongly suggest that Hcy induces damage to neural cells, thereby leading to the manifestation of symptoms resembling depression.

Numerous studies across diverse molecular levels have consistently demonstrated the undeniable importance of synaptic activity in the establishment of neural pathways that underlie cognitive, emotional, and behavioral functions^37^. Prior research has convincingly shown that elevated homocysteine (Hcy) concentrations can lead to a series of negative impacts on synaptic function. Hcy undermines synaptic integrity, disrupts synaptic plasticity, and disturbs neurotransmitter synthesis, release, and recycling, resulting in synaptic dysfunction or injury. For example, Kamat et al. in 2016 found that in Hcy-injected C57BL/6J mice, there was a significant decrease in the expressions of mRNAs and proteins encoding synaptophysin and post - synaptic markers, directly evidencing Hcy’s harmful effects on synaptic structure^38^. Algaidi et al. injected hooded Lister rats with Hcy daily for up to 14 weeks and demonstrated that elevated Hcy levels significantly affected neuronal communication and synaptic transmission in the hippocampus by interfering with long-term potentiation^39^. The synaptic vesicle cycle, vital for normal neurotransmitter release and synaptic communication, when disrupted, harms neurons in multiple ways. It can reduce neurotransmitter release, impair synaptic transmission and neuronal communication, disrupt neural circuits, and potentially cause neurodegeneration^40^. Abnormal cycling also causes the accumulation of misfolded proteins or damaged vesicles in neurons, triggering stress responses like the unfolded protein response, which, if persistent, may lead to neuronal apoptosis^41^. Additionally, it affects membrane component recycling and synaptic structure maintenance, making the synaptic membrane unstable and further contributing to neuronal dysfunction and neurodegeneration^42^. In our present study, we observed that Hcy regulates the expressions of four genes (*snap25*, *cplx1*, *slc32a1*, and *atp6v1e2*) related to the synaptic vesicle cycle in N2a cells through mRNA chip screening, and these findings were further verified in rat brain tissue. Overall, the above - mentioned research indicates that synaptic disorder is a key aspect in understanding the impact of elevated Hcy on neural cells, which not only deepens our understanding of the pathophysiological mechanisms of Hcy-induced neural damage but also offers potential therapeutic targets.

In this study, we successfully identified four genes (*snap25*, *cplx1*, *slc32a1* and *atp6v1e2*) that exhibit differential expression in response to Hcy. These genes are all situated within presynaptic nerve endings and collectively contribute to the regulation of synaptic vesicle exocytosis. It is widely acknowledged that exocytosis represents a crucial step in the synaptic vesicle cycle, necessitating the assembly and disassembly of protein complexes, including the SNARE complex, which is a soluble N-ethylmaleimide-sensitive-factor attachment receptor complex^43^. Previous studies have provided evidence for the pivotal involvement of the protein SNAP25 in the SNARE complex, whereas Complexin is a protein that binds to fully assembled SNARE complexes^44^. Alterations in the expression of *snap25* and *cplx1*, which encode the SNAP25 and Complexin I proteins correspondingly, lead to the depletion of individual SNARE proteins and impede the assembly of SNARE complexes^45^. Additionally, *atp6v1e2* mRNA encodes the V-type proton ATPase subunit E2. V-ATPase interacts with the SNARE complexes implicated in synaptic vesicle docking and exocytosis^46^. Therefore, it seems that the down-regulation of SNAP25, Complexin I and V-ATPase induced by HHcy may contribute to impairments in the formation and functioning of the exocytosis-related SNARE complex within synapses. This inhibition of exocytosis and neurotransmitter release could give rise to impairments in neurotransmission and neurobehavioral dysfunction.

We also observed that the expression of *slc32a1*, which encodes the Vesicular GABA transporter (VGAT), was decreased in N2a cells treated with Hcy. VGAT is a crucial protein involved in the canonical molecular machinery responsible for GABA transmission and vesicular packaging^47^. Its deletion leads to a complete loss of synaptic GABA release^48^. A previous study demonstrated that the knockout of *slc32a1* results in the direct release of GABA in the forebrain cholinergic system^49^. These findings suggest that Hcy may diminish GABA transmission and vesicular packaging by reducing the expression of *slc32a1* mRNA. On the other hand, previous research has indicated that Hcy functions as a GABA-A receptor antagonist by competing with the GABA-A receptor agonist, muscimol, for receptor binding^50^. GABA, an inhibitory neurotransmitter and ligand of the GABA-A receptor, has been shown to alleviate complications related to vascular dementia and stroke^51^. Collectively, the results of our study, along with others, indicate that Hcy may act as an antagonist of GABA and/or directly impact GABA release by reducing the expression of *slc32a1* mRNA, thereby exacerbating dysfunction within the nervous system. Furthermore, the activation of N-methyl-D-aspartate receptors (NMDARs) by Hcy and the resulting intracellular calcium accumulation have been widely recognized as significant contributors to Hcy-induced neurotoxicity^52^. The previous evidence also showed that excessive intracellular calcium signaling leads to decreased expression of VGAT, ultimately exacerbating neuroinflammation in a model of multiple microinfarcts^53^. Based on the aforementioned findings, it can be inferred that the modifications of VGAT caused by Hcy may be attributed to its impact on intracellular calcium ions via NMDARs in this study.

In recent years, increasing attention has been paid to the role of synaptic vesicle dynamics in maintaining neuronal homeostasis. Efficient synaptic transmission is crucial for neuronal survival and the integrity of neural networks. Disruptions in synaptic transmission, particularly in newly formed synaptic connections, can lead to synaptic degradation and induce neuronal apoptosis^54^. The synaptic vesicle cycle plays a key regulatory role in effective neurotransmitter release, and its dysfunction may impair synaptic communication, thereby activating apoptosis-related pathways such as the p53 signaling cascade^55,56^. Animal studies have also shown that alterations in synaptic vesicle dynamics are closely associated with various forms of neuronal apoptosis, including non-classical pathways like parthanatos. Additionally, mitochondrial dysfunction and energy metabolism disturbances are tightly linked to synaptic vesicle turnover, which may further exacerbate synaptic impairment and promote neuronal apoptosis^57,58^. Therefore, these findings suggest that synaptic vesicle cycle dysfunction may play a crucial role in linking synaptic dysfunction and neuronal apoptosis. The findings of our study indicate that Hcy causes neuronal apoptosis in vivo and in vitro. This effect may be attributed to Hcy’s ability to modify the expression of genes associated with the circulation of synaptic vesicles, resulting in impaired synaptic vesicle function. However, the precise mechanism through which Hcy regulates these genes remains elusive. It is notable that homocysteine is a demethylated derivative of methionine, generated during the process of “one-carbon metabolism”. Elevated levels of homocysteine could serve as a clinical biomarker for suboptimal methylation. Hence, it is hypothesized that intervention targeting Hcy may modulate the aberrant methylation patterns of key genes involved in vesicular circulation, thereby potentially explaining the altered expression of Hcy and subsequent neuronal damage. Subsequently, additional investigation is warranted to elucidate the precise mechanism through which Hcy influences the expression of genes involved in the synaptic vesicle cycle.

This study has several limitations. First, while animal and cell models provide valuable insights, they may not fully replicate the complexity of the human nervous system. Second, N2a cells, as a cell line, differ from primary neurons in physiological functions and gene expression, which may affect the interpretation of Hcy-induced neurotoxicity. Third, the study focused on mRNA and protein changes but did not explore epigenetic mechanisms, such as DNA methylation, which could further clarify Hcy’s neurotoxic effects.

## Conclusions

The present study identifies several key genes, namely *snap25*, *cplx1*, *slc32a1*, and *atp6v1e2*, within N2a cells subjected to Hcy treatment, thereby illuminating a novel mechanism wherein Hcy is implicated in various stages of the synaptic vesicle cycle. These discoveries offer fresh perspectives on the molecular mechanisms underlying Hcy-induced neurotoxicity and hold promise for the formulation of novel preventive and therapeutic approaches targeting diseases associated with elevated Hcy levels.

## Electronic supplementary material

Below is the link to the electronic supplementary material.

Supplementary Material 1

## Data Availability

The data that support the findings of this study are available from the corresponding author upon reasonable request.

## Declarations

### ARRIVE guidelines statement

The authors have read the ARRIVE guidelines, and the manuscript was prepared and revised according to the ARRIVE guidelines.
