# Supplementary material for: The involvement of the synaptic vesicle cycle in homocysteine induced neurotoxicity in vitro and in vivo
Source: Sci Rep. 2025 May 29;15:18909. doi: 10.1038/s41598-025-98306-3 (PMC12122915; doi:10.1038/s41598-025-98306-3)
Supplement: Supplementary file 1 — Supplementary Material 1 [file 41598_2025_98306_MOESM1_ESM.pdf]

Figure 4

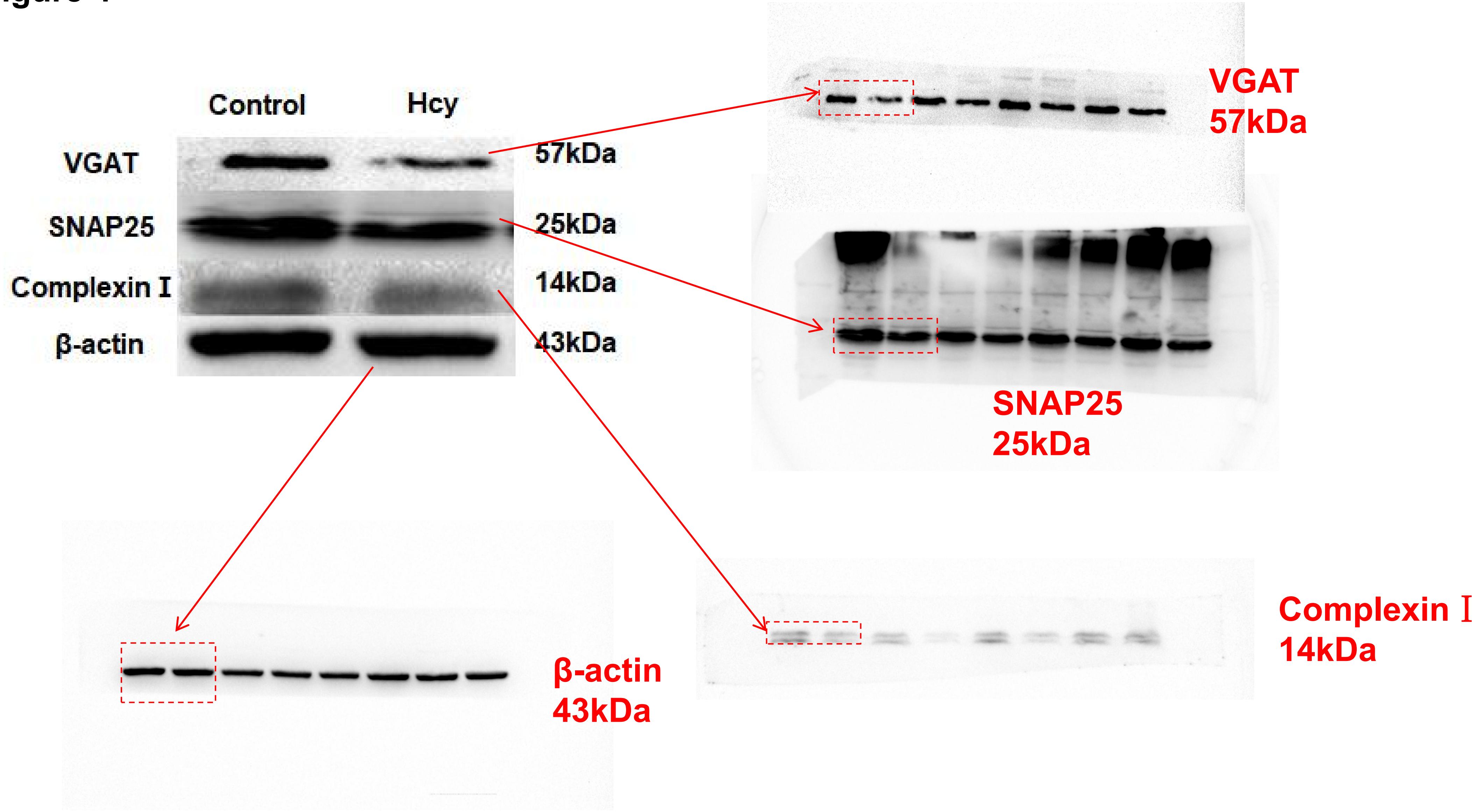

Control
Hcy
VGAT
SNAP25
Complexin I
β-actin
57kDa
25kDa
14kDa
43kDa
VGAT
57kDa
SNAP25
25kDa
β-actin
43kDa
Complexin I
14kDa

Figure 4

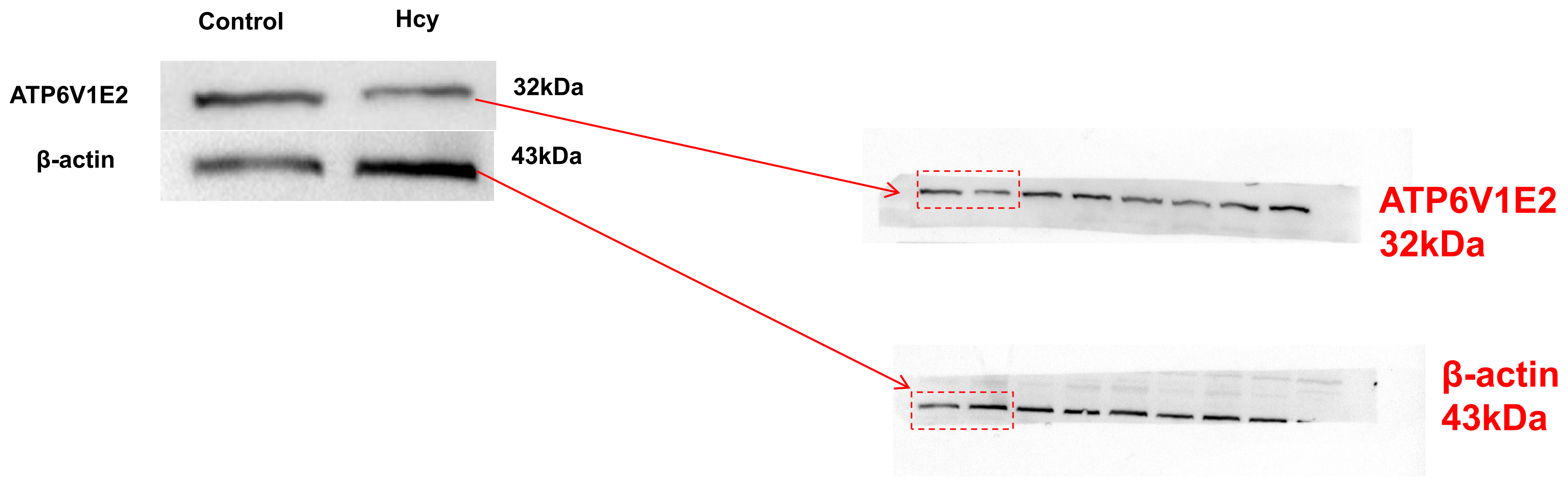

The samples derive from the same experiment or parallel experiments and that gels/blots were processed in parallel.

Figure 5

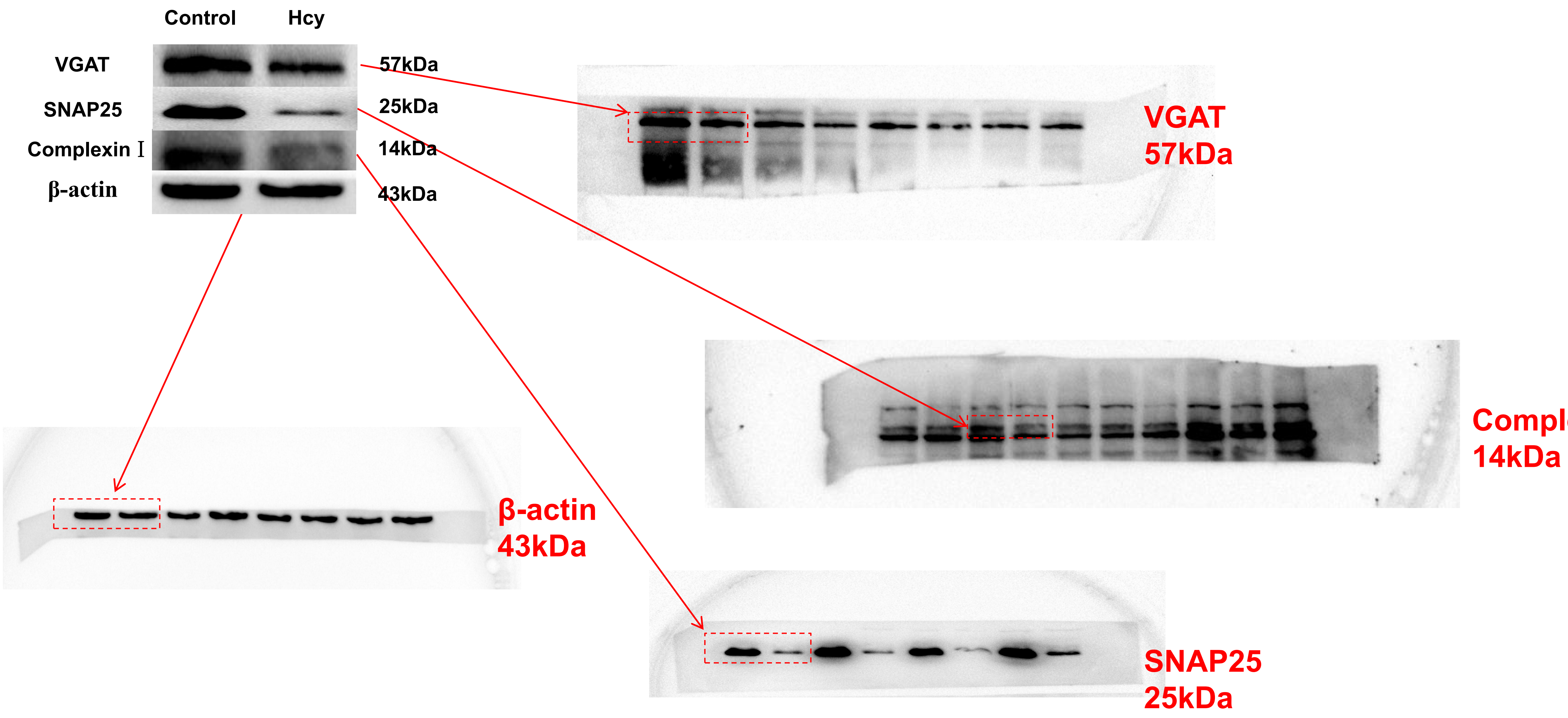

Control
Hcy
VGAT
SNAP25
Complexin Ⅰ
β-actin
57kDa
25kDa
14kDa
43kDa
VGAT
57kDa
Compl
14kDa
β-actin
43kDa
SNAP25
25kDa

Figure 5

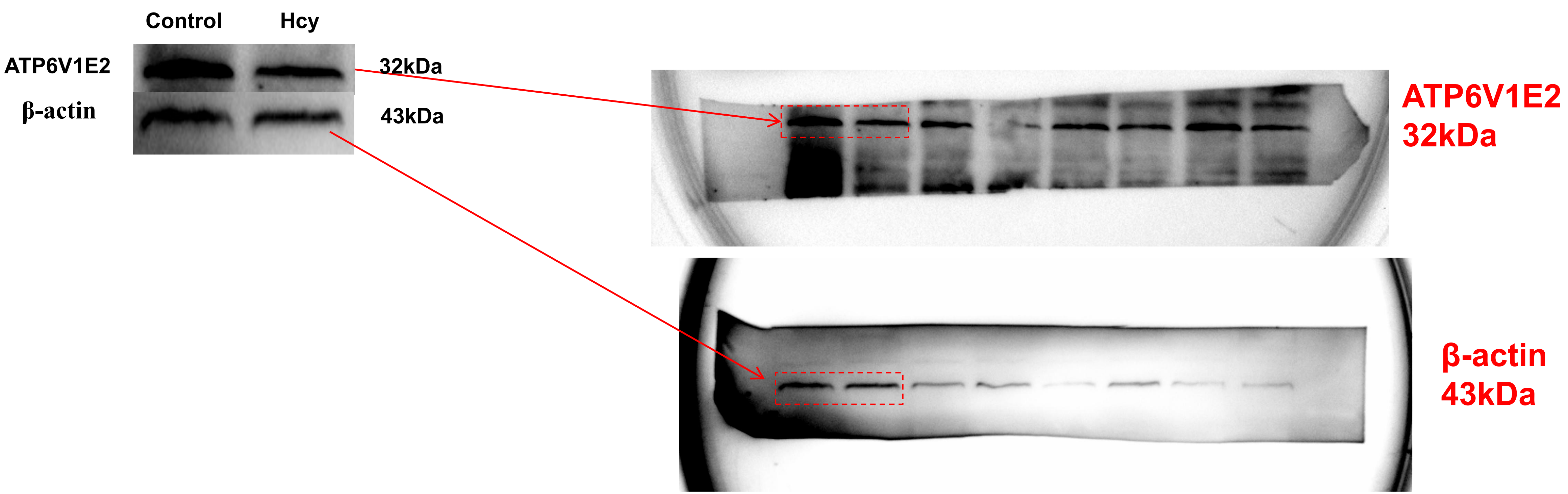

The samples derive from the same experiment or parallel experiments and that gels/blots were processed in parallel.
